# Professor Paul F. Eve

**Published:** 1859-12

**Authors:** 


### PROFESSOR PAUL F. EVE.

This distinguished Professor in the University of Nashville has been making a tour in Europe, where he has written a series of spirited letters to the *Nashville Medical Journal.* Everywhere, at Milan, Turin, Paris, London, Prof. Eve seems to have been received with the attention due to his position. He was elected corresponding member of the Imperial Academy of Medicine of Vienna, and returns, doubtless, with mind enriched and spirit refreshed, to the duties of his course and practice.

---

Dr. S. W. BUTLER, senior editor of the *Medical and Surgical Reporter*, Philadelphia, has been appointed Chief Resident Physician to the Philadelphia Lunatic Asylum.

---

We are pleased to learn that Prof. Blackman, of the Ohio Medical College, has in preparation a work on surgery, intended as a text book for students, and a guide for practitioners, which will be issued from the press in season for the courses of 1860.

Prof. Blackman has many qualifications for the task, among which may be particularly noticed a good knowledge of his profession, an acquaintance with the standard authors, and a mind which will take pleasure in collecting and arranging the scattered and valuable contributions of American surgeons, instead of neglecting, ignoring or garbling them, as is oftener done by writers of systems of surgery. We make this statement principally on the evidence afforded by his notes to Velpeau, by Mott, which afford proofs of his industry and impartiality, and a perusal of his contributions to periodical literature, which are distinguished by careful attention and just appreciation, and in which statistics of operations are frequently presented. We think the appearance of his work will be anticipated with pleasure.

---

We are informed that a new medical journal will soon be issued in Cincinnati, under the editorial management of Professors Lawson and Blackman.

The character and qualifications of these gentlemen are a guarantee that it will be worthy of the best portion of the profession in Cincinnati, which has hitherto been greatly in need of a suitable organ.

---

We learn from the *Boston Medical and Surgical Journal*, that Dr. Eli Ives has declined the office of Junior Secretary of the American Medical Association. President Miller has appointed Dr. Stephen G. Hubbard of New Haven, to fill the place. Dr. Eli Ives is 81 years of age, and the appointment was probably *intended* for Dr. N. B. Ives. It was offered him by the President, but on account of ill health he declined it, and Dr. Hubbard received the appointment.

We never doubted that the profession of New Haven would give the Association a hearty welcome, but when, in announcing that though Connecticut was not represented in the meeting at Louisville, the next was appointed at New Haven, we remarked that the profession of that city would be taken by surprise, one or two of the journals took us to task for intimating such a thing. The above item will show how bunglingly the affair was managed. It would be a little singular if the appointment of an octogenarian as *Junior* Secretary of the Association had not caused a little surprise.

We mark the above extract from the *Medical and Surgical*

*Reporter*, for the purpose of calling attention to a movement initiated at the last meeting of the Illinois State Medical Society, looking to the establishment of a rule that, at future meetings of the National Association, the presidents should *not* be chosen from among the members of the profession of the State or city in which the meetings may be held.

This movement originated, it is understood, with a single ambitious individual, and should it be followed up, may be regarded as a singular additional compliment to the profession of the city of New Haven, and the State of Connecticut.

